# *N*′-[(*E*)-1-(5-Bromo-2-hydroxy­phen­yl)ethyl­idene]benzohydrazide

**DOI:** 10.1107/S1600536808039755

**Published:** 2008-11-29

**Authors:** Chang-Zheng Zheng, Chang-You Ji, Xiu-Li Chang, Li-qin Zhang

**Affiliations:** a College of Environmental and Chemical Engineering, Xi’an Polytechnic University, 710048 Xi’an, Shaanxi, People’s Republic of China; b Department of Materials Science and Chemical Engineering, Sichuan University of Science and Engineering , 643000 Zigong, Sichuan, People’s Republic of China

## Abstract

The C=N double bond in the title compound, C_15_H_13_BrN_2_O_2_, is *trans* *E* configured and the dihedral angle between the aromatic ring planes is 22.3 (1)°. The crystal structure is stabilized by intra­molecular O—H⋯O and inter­molecular N—H⋯O hydrogen bonds.

## Related literature

For aroylhydrazones and their biological activity, see: Singh *et al.* (1982 ▶); Salem (1998 ▶); Carcelli *et al.* (1995 ▶). 
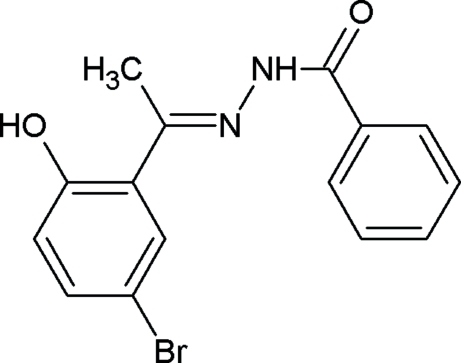


## Experimental

### 

#### Crystal data

C_15_H_13_BrN_2_O_2_
*M* *_r_* = 333.18
Monoclinic, 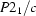

*a* = 7.3761 (15) Å
*b* = 28.270 (6) Å
*c* = 8.6089 (13) Å
β = 116.928 (12)°
*V* = 1600.5 (5) Å^3^
*Z* = 4
Mo *K*α radiation
μ = 2.57 mm^−1^
*T* = 298 (2) K
0.12 × 0.08 × 0.06 mm

#### Data collection

Siemens SMART CCD area-detector diffractometer
Absorption correction: multi-scan (*SADABS*; Sheldrick, 1996 ▶) *T*_min_ = 0.748, *T*_max_ = 0.861
8028 measured reflections
2830 independent reflections
1490 reflections with *I* > 2σ(*I*)
*R*_int_ = 0.062

#### Refinement

*R*[*F* ^2^ > 2σ(*F* ^2^)] = 0.068
*wR*(*F* ^2^) = 0.188
*S* = 1.01
2830 reflections
183 parameters
H-atom parameters constrained
Δρ_max_ = 0.87 e Å^−3^
Δρ_min_ = −0.37 e Å^−3^

### 

Data collection: *SMART* (Siemens, 1996 ▶); cell refinement: *SAINT* (Siemens, 1996 ▶); data reduction: *SAINT*; program(s) used to solve structure: *SHELXS97* (Sheldrick, 2008 ▶); program(s) used to refine structure: *SHELXL97* (Sheldrick, 2008 ▶); molecular graphics: *SHELXTL* (Sheldrick, 2008 ▶); software used to prepare material for publication: *SHELXTL*.

## Supplementary Material

Crystal structure: contains datablocks I, global. DOI: 10.1107/S1600536808039755/bt2814sup1.cif

Structure factors: contains datablocks I. DOI: 10.1107/S1600536808039755/bt2814Isup2.hkl

Additional supplementary materials:  crystallographic information; 3D view; checkCIF report

## Figures and Tables

**Table 1:** Hydrogen-bond geometry (Å, °)

*D*—H⋯*A*	*D*—H	H⋯*A*	*D*⋯*A*	*D*—H⋯*A*
O1—H1⋯N1	0.82	1.85	2.522 (6)	138
N2—H2⋯O2^i^	0.86	2.14	2.889 (6)	146

Symmetry code: (i) 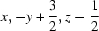.

**Fig. 1.**
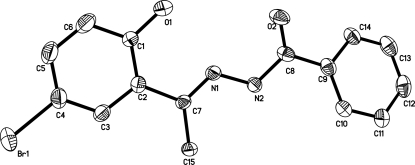
The molecular structure the title compound showing the atom-numbering scheme. Displacement ellipsoids are drawn at the 30% probability level.

**Fig. 2.**
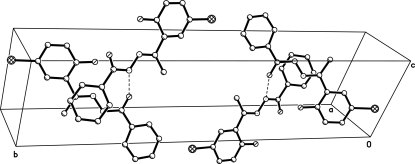
The crystal packing of the title compound. Dashed lines show hydrogen bonds.

## supplementary crystallographic information

### Comment

The chemistry of aroylhydrazones continues to attract much attention due to their coordination ability to metal ions (Singh *et al.*, 1982; Salem, 1998) and their biological activity (Singh *et al.*, 1982; Carcelli *et al.*, 1995). As an extension of work on the structural characterization of aroylhydrazone derivatives, the title compound was synthesized and its crystal structure is reported here.

The title molecule displays a *trans* configured C=N double bond (Fig. 1). The crystal structure is stabilized by intramolecular O—H···O and intermolecular N—H···O hydrogen bonds (Table 1. and Fig. 2).

### Experimental

Benzoylhydrazine (0.02 mol, 2.72 g) was dissolved in anhydrous ethanol (50 ml), and 1-(5-bromo-2-hydroxyphenyl)ethanone (0.02 mol, 4.30 g) was added. The reaction mixture was refluxed for 6 h with stirring, then the resulting precipitate was collected by filtration, washed several times with ethanol and dried *in vacuo* (yield 85%). The compound (2.0 mmol, 0.67 g) was dissolved in dimethylformamide (30 ml) and kept at room temperature for 30 d to obtain yellow single crystals suitable for X-ray diffraction.

### Refinement

All H atoms were positioned geometrically and treated as riding on their parent atoms,with *C*—*H*(methyl) = 0.96 Å, C—H(aromatic) = 0.93 Å, O—H = 0.82 Å, and N—H = 0.86 Å and with *U*_iso_(H) =1.5*U*_eq_(C_methyl_,*O*) and 1.2*U*_eq_(C_aromatic_,*N*).

### Crystal data

**Table:**

C_15_H_13_BrN_2_O_2_	*F*_000_ = 672
*M**_r_* = 333.18	*D*_x_ = 1.383 Mg m^−^^3^
Monoclinic, *P*2_1_/*c*	Mo *K*α radiation λ = 0.71073 Å
Hall symbol: -P 2ybc	Cell parameters from 1189 reflections
*a* = 7.3761 (15) Å	θ = 2.9–20.7º
*b* = 28.270 (6) Å	µ = 2.57 mm^−^^1^
*c* = 8.6089 (13) Å	*T* = 298 (2) K
β = 116.928 (12)º	Block, yellow
*V* = 1600.5 (5) Å^3^	0.12 × 0.08 × 0.06 mm
*Z* = 4	

### Data collection

**Table:**

Siemens SMART CCD area-detector diffractometer	2830 independent reflections
Radiation source: fine-focus sealed tube	1490 reflections with *I* > 2σ(*I*)
Monochromator: graphite	*R*_int_ = 0.062
*T* = 298(2) K	θ_max_ = 25.1º
φ and ω scans	θ_min_ = 3.0º
Absorption correction: multi-scan(SADABS; Sheldrick, 1996)	*h* = −8→8
*T*_min_ = 0.748, *T*_max_ = 0.861	*k* = −33→33
8028 measured reflections	*l* = −7→10

### Refinement

**Table:**

Refinement on *F*^2^	Secondary atom site location: difference Fourier map
Least-squares matrix: full	Hydrogen site location: inferred from neighbouring sites
*R*[*F*^2^ > 2σ(*F*^2^)] = 0.068	H-atom parameters constrained
*wR*(*F*^2^) = 0.188	*w* = 1/[σ^2^(*F*_o_^2^) + (0.089*P*)^2^ + 0.7896*P*] where *P* = (*F*_o_^2^ + 2*F*_c_^2^)/3
*S* = 1.01	(Δ/σ)_max_ = 0.002
2830 reflections	Δρ_max_ = 0.87 e Å^−^^3^
183 parameters	Δρ_min_ = −0.37 e Å^−^^3^
Primary atom site location: structure-invariant direct methods	Extinction correction: none

### Special details

**Table:**

Geometry. All e.s.d.'s (except the e.s.d. in the dihedral angle between two l.s. planes)are estimated using the full covariance matrix. The cell e.s.d.'s are takeninto account individually in the estimation of e.s.d.'s in distances, anglesand torsion angles; correlations between e.s.d.'s in cell parameters are onlyused when they are defined by crystal symmetry. An approximate (isotropic)treatment of cell e.s.d.'s is used for estimating e.s.d.'s involving l.s. planes.
Refinement. Refinement of *F*^2^ against ALL reflections. The weighted *R*-factor *wR* and goodness of fit *S* are based on *F*^2^, conventional *R*-factors *R* are based on *F*, with *F* set to zero for negative *F*^2^. The threshold expression of *F*^2^ > σ(*F*^2^) is used only for calculating *R*-factors(gt) *etc*. and is not relevant to the choice of reflections for refinement. *R*-factors based on *F*^2^ are statistically about twice as large as those based on *F*, and *R*- factors based on ALL data will be even larger.

### Fractional atomic coordinates and isotropic or equivalent isotropic displacement parameters (Å^2^)

**Table:**

	*x*	*y*	*z*	*U*_iso_*/*U*_eq_	
Br1	0.81952 (14)	1.00196 (2)	0.69361 (11)	0.0935 (4)	
O1	0.6647 (7)	0.79484 (14)	0.7321 (5)	0.0613 (11)	
H1	0.6183	0.7820	0.6365	0.092*	
O2	0.3297 (6)	0.70963 (13)	0.4419 (5)	0.0602 (11)	
N1	0.4384 (6)	0.79657 (14)	0.4079 (5)	0.0391 (10)	
N2	0.3180 (6)	0.76994 (15)	0.2660 (5)	0.0416 (10)	
H2	0.2720	0.7810	0.1620	0.050*	
C1	0.6935 (8)	0.84139 (19)	0.7143 (6)	0.0442 (13)	
C2	0.6016 (8)	0.86503 (17)	0.5556 (6)	0.0390 (12)	
C3	0.6426 (8)	0.91292 (19)	0.5540 (7)	0.0497 (14)	
H3	0.5829	0.9294	0.4491	0.060*	
C4	0.7677 (8)	0.9363 (2)	0.7018 (8)	0.0555 (15)	
C5	0.8592 (10)	0.9132 (2)	0.8592 (8)	0.0655 (17)	
H5	0.9461	0.9293	0.9598	0.079*	
C6	0.8202 (9)	0.8661 (2)	0.8654 (8)	0.0658 (18)	
H6	0.8789	0.8503	0.9716	0.079*	
C7	0.4633 (7)	0.84066 (17)	0.3902 (6)	0.0370 (12)	
C8	0.2737 (8)	0.72504 (18)	0.2962 (7)	0.0430 (13)	
C9	0.1524 (8)	0.69622 (19)	0.1385 (7)	0.0460 (13)	
C10	0.0099 (8)	0.7153 (2)	−0.0181 (7)	0.0509 (14)	
H10	−0.0132	0.7477	−0.0273	0.061*	
C11	−0.0978 (9)	0.6865 (2)	−0.1603 (8)	0.0581 (16)	
H11	−0.1931	0.6994	−0.2649	0.070*	
C12	−0.0621 (10)	0.6383 (2)	−0.1449 (9)	0.0641 (17)	
H12	−0.1335	0.6186	−0.2400	0.077*	
C13	0.0790 (11)	0.6191 (2)	0.0110 (9)	0.0618 (16)	
H13	0.1027	0.5866	0.0200	0.074*	
C14	0.1832 (9)	0.64743 (19)	0.1507 (7)	0.0496 (14)	
H14	0.2760	0.6342	0.2557	0.060*	
C15	0.3682 (9)	0.86765 (19)	0.2249 (6)	0.0535 (15)	
H15A	0.4673	0.8733	0.1836	0.080*	
H15B	0.3186	0.8973	0.2445	0.080*	
H15C	0.2571	0.8498	0.1395	0.080*	

### Atomic displacement parameters (Å^2^)

**Table:**

	*U*^11^	*U*^22^	*U*^33^	*U*^12^	*U*^13^	*U*^23^
Br1	0.1011 (7)	0.0462 (5)	0.0962 (7)	−0.0133 (4)	0.0123 (5)	−0.0172 (4)
O1	0.073 (3)	0.058 (3)	0.040 (2)	−0.003 (2)	0.014 (2)	0.0092 (19)
O2	0.103 (3)	0.040 (2)	0.041 (2)	0.000 (2)	0.037 (2)	0.0038 (17)
N1	0.051 (3)	0.036 (3)	0.029 (2)	0.002 (2)	0.0173 (19)	−0.0012 (19)
N2	0.054 (3)	0.037 (3)	0.032 (2)	−0.006 (2)	0.019 (2)	−0.0022 (19)
C1	0.043 (3)	0.045 (3)	0.037 (3)	−0.003 (2)	0.011 (2)	0.004 (2)
C2	0.034 (3)	0.039 (3)	0.044 (3)	0.002 (2)	0.017 (2)	−0.005 (2)
C3	0.047 (3)	0.049 (3)	0.040 (3)	0.008 (3)	0.009 (3)	−0.005 (3)
C4	0.047 (3)	0.048 (4)	0.062 (4)	0.001 (3)	0.015 (3)	−0.014 (3)
C5	0.064 (4)	0.067 (4)	0.051 (4)	−0.005 (3)	0.013 (3)	−0.020 (3)
C6	0.062 (4)	0.079 (5)	0.040 (3)	0.002 (3)	0.008 (3)	−0.003 (3)
C7	0.044 (3)	0.037 (3)	0.028 (3)	0.004 (2)	0.014 (2)	−0.001 (2)
C8	0.064 (4)	0.032 (3)	0.041 (3)	0.006 (3)	0.031 (3)	0.000 (2)
C9	0.057 (3)	0.043 (3)	0.049 (3)	−0.009 (3)	0.033 (3)	−0.004 (3)
C10	0.057 (3)	0.040 (3)	0.053 (4)	−0.006 (3)	0.022 (3)	0.004 (3)
C11	0.058 (4)	0.052 (4)	0.055 (4)	−0.013 (3)	0.017 (3)	−0.006 (3)
C12	0.070 (4)	0.062 (4)	0.064 (4)	−0.024 (3)	0.034 (4)	−0.027 (3)
C13	0.082 (4)	0.041 (3)	0.079 (5)	−0.009 (3)	0.050 (4)	−0.011 (3)
C14	0.066 (4)	0.036 (3)	0.053 (3)	−0.004 (3)	0.033 (3)	−0.003 (3)
C15	0.071 (4)	0.036 (3)	0.034 (3)	0.001 (3)	0.006 (3)	0.002 (2)

### Geometric parameters (Å, °)

**Table:**

Br1—C4	1.902 (6)	C6—H6	0.9300
O1—C1	1.353 (6)	C7—C15	1.482 (7)
O1—H1	0.8200	C8—C9	1.485 (7)
O2—C8	1.210 (6)	C9—C10	1.389 (7)
N1—C7	1.279 (6)	C9—C14	1.394 (7)
N1—N2	1.366 (5)	C10—C11	1.382 (7)
N2—C8	1.365 (6)	C10—H10	0.9300
N2—H2	0.8600	C11—C12	1.384 (8)
C1—C2	1.391 (7)	C11—H11	0.9300
C1—C6	1.397 (8)	C12—C13	1.383 (9)
C2—C3	1.389 (7)	C12—H12	0.9300
C2—C7	1.492 (7)	C13—C14	1.358 (8)
C3—C4	1.358 (7)	C13—H13	0.9300
C3—H3	0.9300	C14—H14	0.9300
C4—C5	1.376 (8)	C15—H15A	0.9600
C5—C6	1.367 (9)	C15—H15B	0.9600
C5—H5	0.9300	C15—H15C	0.9600
			
C1—O1—H1	109.5	O2—C8—C9	122.1 (5)
C7—N1—N2	120.1 (4)	N2—C8—C9	115.7 (4)
C8—N2—N1	117.2 (4)	C10—C9—C14	118.9 (5)
C8—N2—H2	121.4	C10—C9—C8	123.5 (5)
N1—N2—H2	121.4	C14—C9—C8	117.6 (5)
O1—C1—C2	123.2 (4)	C11—C10—C9	120.7 (6)
O1—C1—C6	117.0 (5)	C11—C10—H10	119.6
C2—C1—C6	119.8 (5)	C9—C10—H10	119.6
C3—C2—C1	117.8 (5)	C10—C11—C12	119.1 (6)
C3—C2—C7	119.9 (4)	C10—C11—H11	120.4
C1—C2—C7	122.3 (5)	C12—C11—H11	120.4
C4—C3—C2	121.6 (5)	C13—C12—C11	120.4 (5)
C4—C3—H3	119.2	C13—C12—H12	119.8
C2—C3—H3	119.2	C11—C12—H12	119.8
C3—C4—C5	120.9 (6)	C14—C13—C12	120.2 (6)
C3—C4—Br1	120.2 (5)	C14—C13—H13	119.9
C5—C4—Br1	118.9 (4)	C12—C13—H13	119.9
C6—C5—C4	118.9 (6)	C13—C14—C9	120.7 (6)
C6—C5—H5	120.6	C13—C14—H14	119.7
C4—C5—H5	120.6	C9—C14—H14	119.7
C5—C6—C1	121.0 (6)	C7—C15—H15A	109.5
C5—C6—H6	119.5	C7—C15—H15B	109.5
C1—C6—H6	119.5	H15A—C15—H15B	109.5
N1—C7—C15	125.8 (4)	C7—C15—H15C	109.5
N1—C7—C2	114.2 (4)	H15A—C15—H15C	109.5
C15—C7—C2	120.0 (5)	H15B—C15—H15C	109.5
O2—C8—N2	122.2 (5)		
			
C7—N1—N2—C8	−170.7 (5)	C1—C2—C7—N1	−0.3 (7)
O1—C1—C2—C3	179.8 (5)	C3—C2—C7—C15	−0.6 (7)
C6—C1—C2—C3	0.8 (8)	C1—C2—C7—C15	179.6 (5)
O1—C1—C2—C7	−0.4 (8)	N1—N2—C8—O2	3.3 (7)
C6—C1—C2—C7	−179.4 (5)	N1—N2—C8—C9	−176.6 (4)
C1—C2—C3—C4	−0.2 (8)	O2—C8—C9—C10	149.0 (5)
C7—C2—C3—C4	−180.0 (5)	N2—C8—C9—C10	−31.1 (7)
C2—C3—C4—C5	0.2 (9)	O2—C8—C9—C14	−30.3 (8)
C2—C3—C4—Br1	−180.0 (4)	N2—C8—C9—C14	149.6 (5)
C3—C4—C5—C6	−0.8 (9)	C14—C9—C10—C11	−0.8 (8)
Br1—C4—C5—C6	179.3 (5)	C8—C9—C10—C11	179.9 (5)
C4—C5—C6—C1	1.5 (10)	C9—C10—C11—C12	0.0 (8)
O1—C1—C6—C5	179.4 (6)	C10—C11—C12—C13	0.3 (9)
C2—C1—C6—C5	−1.5 (9)	C11—C12—C13—C14	0.3 (9)
N2—N1—C7—C15	1.2 (8)	C12—C13—C14—C9	−1.2 (9)
N2—N1—C7—C2	−178.9 (4)	C10—C9—C14—C13	1.4 (8)
C3—C2—C7—N1	179.5 (5)	C8—C9—C14—C13	−179.3 (5)

### Hydrogen-bond geometry (Å, °)

**Table:**

*D*—H···*A*	*D*—H	H···*A*	*D*···*A*	*D*—H···*A*
O1—H1···N1	0.82	1.85	2.522 (6)	138
N2—H2···O2^i^	0.86	2.14	2.889 (6)	146

Symmetry codes: (i) *x*, −*y*+3/2, *z*−1/2.
